# Stem cells from human amniotic fluid exert immunoregulatory function *via* secreted indoleamine 2,3-dioxygenase1

**DOI:** 10.1111/jcmm.12534

**Published:** 2015-03-17

**Authors:** Rita Romani, Irene Pirisinu, Mario Calvitti, Maria Teresa Pallotta, Marco Gargaro, Giovanni Bistoni, Carmine Vacca, Alessandro Di Michele, Ciriana Orabona, Jessica Rosati, Matteo Pirro, Stefano Giovagnoli, Davide Matino, Paolo Prontera, Gabriella Rosi, Ursula Grohmann, Vincenzo N Talesa, Emilio Donti, Paolo Puccetti, Francesca Fallarino

**Affiliations:** a Department of Experimental Medicine, University of PerugiaPerugia, Italy; b Plastic Surgery Unit, Hospital Universitario de la RiberaValencia, Spain; c Department of Surgery, ‘La Sapienza’ UniversityRome, Italy; d Department of Physics and Geology, University of PerugiaPerugia, Italy; e iPS-Cellular Reprogramming Unit, Fondazione Casa Sollievo della Sofferenza, MendelRome, Italy; f Department of Medicine, University of PerugiaPerugia, Italy; g Department of Pharmaceutical Sciences, University of PerugiaPerugia, Italy; 8 Department of Surgery and Biomedical Sciences, University of PerugiaPerugia, Italy

**Keywords:** cell culture, pluripotent stem cells, immunosuppression, lymphocytes, cloning, T cells

## Abstract

Although human amniotic fluid does contain different populations of foetal-derived stem cells, scanty information is available on the stemness and the potential immunomodulatory activity of *in vitro* expanded, amniotic fluid stem cells. By means of a methodology unrequiring immune selection, we isolated and characterized different stem cell types from second-trimester human amniotic fluid samples (human amniotic fluid stem cells, HASCs). Of those populations, one was characterized by a *fast* doubling time, and cells were thus designated as fHASCs. Cells maintained their original phenotype under prolonged *in vitro* passaging, and they were able to originate embryoid bodies. Moreover, fHASCs exhibited regulatory properties when treated with interferon (IFN)-γ, including induction of the immunomodulatory enzyme indoleamine 2,3-dioxygenase 1 (IDO1). On coculture with human peripheral blood mononuclear cells, IFN-γ–treated fHASCs caused significantly decreased T-cell proliferation and increased frequency in CD4^+^ CD25^+^ FOXP3^+^ regulatory T cells. Both effects required an intact IDO1 function and were cell contact-independent. An unprecedented finding in our study was that purified vesicles from IFN-γ–treated fHASCs abundantly expressed the functional IDO1 protein, and those vesicles were endowed with an fHASC-like regulatory function. *In vivo*, fHASCs were capable of immunoregulatory function, promoting allograft survival in a mouse model of allogeneic skin transplantation. This was concurrent with the expansion of CD4^+^ CD25^+^ Foxp3^+^ T cells in graft-draining lymph nodes from recipient mice. Thus fHASCs, or vesicles thereof, may represent a novel opportunity for immunoregulatory maneuvers both *in vitro* and *in vivo*.

## Introduction

Foetal cells have been identified in human amniotic fluid and have long been used in prenatal genetic screening 1–3. Since their isolation, it was recognized that at least two types of stem cell are isolated from amniotic fluid, namely, mesenchymal stem cells (AFMSCs) 4,5 and more recently, amniotic fluid stem cells (AFSCs) 6. AFMSCs exhibit the typical characteristics of mesenchymal stromal cells (MSCs), including a fibroblast-like morphology, clonogenic capacity, multipotence, immunosuppressive properties and expression of the membrane-associated antigens and genes identified in MSCs 5–7. In contrast, AFSCs are pluripotent 8–10, and they share characteristics with both human embryonic stem cells (ESCs) and adult stem cells (ASCs) 10. They can be expanded extensively, without feeders, for several generations *in vitro*, maintaining an undifferentiated phenotype. AFSCs do not form tumours when transplanted into immunodeficient mice 6,10,11. Moreover, human AFSCs express the stage-specific embryonic antigen (SSEA)-4, also found in ESCs, and they likewise express the transcription factor OCT-4, which has been associated in ESCs with pluripotency and the maintenance of an undifferentiated state 12. Under specific inducing conditions, clones of AFSCs give rise to lineages representative of the three embryonic germ layers 13. Those properties suggest that AFSCs are distinct from both pluripotent ESCs and multipotential ASCs, likely representing a novel class of stem cells 6,11. Human AFSCs could thus be considered as a potential source of stem cells exempt from most ethical concerns typically associated with the use of ESCs.

Besides sharing properties with MSCs, AFSCs exhibit increased potency and potent expansion capacity 6. Similar to MSCs, AFSCs possess immunoregulatory properties 4,14,15. The immunoregulatory properties of MSCs in inflammatory contexts occur, reportedly, *via* suppression of a variety of inflammatory cytokines and factors released by immune cells at the site of inflammation, including interferon (IFN)-γ, tumour necrosis factor (TNF)-α and interleukin (IL)-1α/β 16. The paracrine mechanisms responsible for MSC effects on the local immune microenvironment include a broad variety of molecular pathways 17–20, among which is the kynurenine pathway of tryptophan degradation mediated by indoleamine 2,3-dioxygenase 1 21.

Indoleamine 2,3-dioxygenase 1 (IDO1), a ‘metabolic’ enzyme conserved through the last 600 million years of evolution, suppresses T-cell responses and promotes fetomaternal tolerance in the mammalian pregnancy 22, and it also exerts regulatory functions in autoimmune 23 and inflammatory settings 24. Its regulation, as well as its mechanisms of action as an immune regulator are composite 25. By tryptophan starvation and kynurenine production, IDO1 activity results in an arrest in T-cell proliferation, induction of T-helper type-1 cell apoptosis, reversible impairment of effector T-cell activity and induction/activation of regulatory T (Treg) cells 26. IDO1 also acts as a tolerogenic signalling molecule in dendritic cells, and is capable of affecting gene transcription 27. Any contribution of IDO1 to the immunoregulatory properties of human amniotic fluid stem cells (HASCs) has not been investigated yet.

In this study, we isolated and characterized HASCs *via* the secondary use of prenatal diagnostic material, employing a novel methodology with no need for immune selection. Fast-growing fHASCs exhibited *in vitro* immunomodulatory properties contingent on IDO1, and they promoted allograft survival in an experimental model. Of particular interest, fHASCs released vesicles that mimicked the regulatory function of whole fHASCs. These findings indicate that fHASCs, and soluble products thereof, may represent a novel type of stem cell material from amniotic fluid, holding promise for both regenerative medicine and modulation of the immune system.

## Material and methods

### HASC Isolation and culture

**Figure 1:**
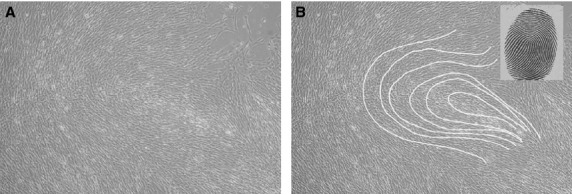
fHASC morphology and shape of the colonies. (A) fHASCs with fibroblast-like morphology; (B) colony shape is similar to dermatoglyphics (inset). Representative data of one from five different lines.

Human amniotic fluid stem cells were obtained from human amniotic fluids of 16–17-week pregnant women (aged 35–40 years), who underwent amniocentesis during routine prenatal diagnosis. The study was approved by the University of Perugia Bioethics Committee, and each participant provided informed consent for the secondary use of amniotic fluid samples. This procedure of stem cell isolation could be applied on fresh amniotic fluid or residual cells from prenatal diagnosis. Briefly, an aliquot (3–5 ml) of fresh amniotic fluid or residual cells from prenatal diagnosis tests was centrifuged to remove either the amniotic fluid or the residual cell culture media. The cell pellet was then plated into flasks and cultured in 4 ml of 18% CHANG B plus 2% CHANG C media (Irvine Scientific, Newtownmountkennedy, Ireland, UK) for 6–7 days. At this time, adherent cells appeared to form colonies (Fig.1). The same selection procedure was applied on residual cells from the prenatal diagnostic procedure. After this first round of cell culture, stem cell isolation consisted of selecting cultures containing cells with a mostly fibroblast-like morphology and a colony shape similar to dermatoglyphics (Fig.1B). The selected colonies were cultured in MSCGM medium (Lonza, Gaithersburg, MD, USA), and medium was replaced every 3–5 days for several passages *in vitro*. The resulting HASC cultures appeared homogeneous after at least three passages *in vitro*.

### Cell growth curves, determination of doubling time, cloning assay and cryopreservation

Human amniotic fluid stem cells were trypsinized at the fifth passage and plated at 10^4^ cells/well (in 12-well plates), in triplicate, and in the presence of MSCGM media. Cells were trypsinized and counted daily for 5 days. The HASC population-doubling time (TD) was calculated by the formula, TD = *t* × log_2_/log (*N*_*t*_/*N*_0_), where *N*_0_ is the inoculum cell number and *N*_*t*_ is the cell harvest number at time *t* 28. Three independent experiments were performed, each in triplicate. To determine any clonogenic capacity, HASCs were cloned by limiting dilution. Briefly, primary plate-adhering HASCs were trypsinized, diluted to 0.5 cells/ml and seeded into 24-well plates in MSCGM and incubated at 37°C with 5% humidified CO_2_. The HASC clones were expanded for ten passages and subsequently evaluated for their immunophenotype and expression of pluripotency markers. Aliquots of 1 × 10^6^ cells were stored in cryovials at −80°C for 3 days, then in liquid nitrogen. The cryoprotectant solution consisted of MSCGM with 50% ES-FBS (Life Technologies, Monza, Italy) and 10% DMSO (Sigma-Aldrich, Saint Louis, MO, USA).

### Flow cytometry

Phenotypical characterization of HASCs was performed as follows. Staining and analysis with FITC- or PE-conjugated antibodies were performed according to standard protocols, on an EPICS flow cytometer, by the use of EXPO 32 ADC software (Beckman Coulter, Pasadena, CA, USA). For HASC surface antigen analysis, cells were harvested by trypsinization, washed with phosphate-buffered saline supplemented with foetal bovine serum (FBS) (3%) and stained with antibodies to the following—HLA A,B,C (Beckton Dickinson, Pasadena, CA, USA); class II DR, CD10, CD11b, CD14, CD29, CD34, CD38, CD44, CD49d, CD73, CD105 (Immunotools, Friesoythe, Germany); CD90, CD117 (c-kit), SSEA3, SSEA4, OCT4 (Biolegend, San Diego, CA, USA). To detect intracellular markers, cells were permeabilized with 0.2% (vol/vol) Triton-X (Sigma-Aldrich) for 30 min. at room temperature before staining.

### RT-PCR analysis

Total RNA was extracted from HASCs by Trizol (Invitrogen, Life Technologies, Monza, Italy) and used as a template for reverse transcription to cDNA. The cDNA was obtained by the RevertAid First Strand cDNA Synthesis Kit (Fermentas, Rodano, Milan, Italy); mRNA quantitation was performed by RT-PCR analyses using Brilliant SYBR Green QPCR Master Mix 2x (Stratagene, Santa Clara, CA, USA) with the Mx3000P qPCR System (Stratagene). The reaction was performed following the manufacturer’s recommendations, using 200 nM of each primer. Each sample was normalized to β-actin (Invitrogen). Final results, expressed as relative expressions, were calculated by MxPro software (Stratagene). Each experiment was repeated at least three times for all cell lines at different passages. The 2^−ΔΔCT^ method was applied as a comparative method of quantitation, and data were normalized to β-actin RNA expression. Primers sequences are shown in Table S1.

### Induced pluripotent stem cell generation

Fibroblasts from healthy donors were nucleofected with pCLXE-hOct3/4-shp53, pCLXE-hSox2-Klf4, pCLXE-hLmyc-Lin28 and pCLXE-GFP episomal vectors (Addgene plasmids 27077, 27078, 27080 and 27082 respectively). After 1 week from nucleofection, fibroblasts were splitted on MEF feeder in induced pluripotent stem cell (iPSC) medium containing 80% DMEM/F12, 20% knockout serum replacement, 10 ng/ml bFGF, 1 mM glutamine, 0.1 mM β-mercaptoethanol, 0.5% penicillin and streptomycin and 1% non-essential amino acid solution. Four-to-six weeks later, iPSC-like colonies were handpicked and amplified.

### Embryoid body and teratoma formation assays

Embryoid body (EB) formation was assessed by the method described by Valli *et al*. 13. For teratoma formation, 5 × 10^6^ HASCs were resuspended in PBS and injected subcutaneously into 8-week-old immunodeficient NOD/SCID mice. Mice were maintained under specific pathogen-free conditions and monitored for tumour formation weekly for a period of 160 days after cell transfer.

### Osteogenic and adipogenic differentiation

To induce osteogenic differentiation, HASCs, harvested at passage #5 and with 60–70% confluency, were cultured in Differentation Media BulletKits-Osteogenic (Lonza), according to manufacturer’s instructions. The differentiation potential for osteogenesis was assessed by mineralization of calcium accumulation on von Kossa staining (Abcam, Bristol, UK), according to producer’s instructions; moreover, we determined changes in RT-PCR expression of specific genes, namely, Secreted Phospho-Protein 1, Bone Gamma-carboxyglutamate (Gla) Protein (BGLAP), Runt-related transcription factor 2 (RUNX2), Alkaline Phosphatase, Liver/bone/kidney (ALPL). To induce adipogenic differentiation, HASCs harvested as indicated above were cultured in Differentiation Media BulletKits-Adipogenic (Lonza). The potential for adipogenic differentiation was assessed by Sudan III staining (Sigma-Aldrich), according to the manufacturer’s instructions. Changes in the expression of specific genes, markers of adipogenic differentiation, such as Peroxisome Proliferator-Activated Receptor Gamma (PPARG), Lipo-Protein Lipase (LPL), Fatty Acid Binding Protein 4 (FABP4), were also determined by RT-PCR.

### Western blot analysis and kynurenine assay

Western blot analysis was performed according to standard procedures. Human IDO1 expression was detected by sequential immunoblotting with anti-IDO1 (10.1) (Millipore, Milan, Italy) and anti–β-tubulin (Sigma-Aldrich) for normalization. IDO1 functional activity was measured *in vitro* in terms of the ability to metabolize tryptophan to l-kynurenine, whose concentrations were measured by high-performance liquid chromatography 29. Briefly, fHASC-derived nanovesicles (NVs) or fHASCs (treated or not with IFN-γ for 24 hrs) were washed and resuspended in medium containing 100 μM tryptophan (Sigma-Aldrich) and then incubated for 4 hrs at 37°C. After incubation, the supernatant was collected and stored at −80°C for quantitation of kynurenine by HPLC. IDO1 activity was expressed as kynurenine concentration (μmol/l) in each sample 29.

### *IDO1* silencing

For *IDO1* silencing, specific siRNA were predesigned on the basis of the respective gene sequence and synthesized by Ambion, Monza, Italy, which also supplied the Negative Control siRNA, and specificity was confirmed using an ON-TARGET*plus* siRNA synthesized by Thermo Scientific (Dharmacon RNAi Technologies, Rodano, Milan, Italy) to exclude potential off-target effects. Transfection of fHASCs was carried out as previously described 30.

### fHASC-PBMC cocultures and FOXP3 expression

For peripheral blood mononuclear cell (PBMC)-fHASC cocultures, PBMCs (3 × 10^5^/well) were activated with 5 μg/ml anti-CD3 mAb (clone OKT3) and then cocultured for 7 days with fHASCs that had been pre-treated or not with 1000 U/ml IFN-γ for 24 hrs. In some experiments, the PBMCs were incubated with NVs isolated from 1 × 10^6^ fHASCs that had been cultured *in vitro* for 2 days. The expression of FOXP3 was evaluated by FACS. Briefly, cells were treated with rat anti-CD16/32 (2.4G2) for 30 min. at 4°C for blocking of Fc receptors before assaying on an LSRFortessa (BD BioSciences, Franklin Lakes, NJ, USA) flow cytometer and analysed by flowJo data analysis software. The following fluorochrome-conjugated mAbs were used, CD4 (RPA-T4) and FOXP3 (150D) (Biolegend). For assessment of any cell–cell contact requirement, a 6.5 mm Transwell system (Corning, Union City, CA, USA) with a 0.4 μm pore polycarbonate membrane insert was used to separate PBMCs (upper chamber) from fHASCs (lower chamber). fHASCs at different PBMC-to-HASC ratios were added to the lower chamber, either as such or after treatment with IFN-γ or transfection with *IDO1* siRNA or negative control siRNA. After 24 hrs, medium was removed and replaced with fresh medium alone. PBMCs (3 × 10^5^) were added to the upper chamber, and recovered at specific times, as detailed in the relevant figure legends.

### Nanovesicle isolation and characterization

Serum-free medium from cultured fHASCs that had been stimulated or not with IFN-γ was pooled and centrifuged at 300 × g for 10 min. to remove intact cells, followed by centrifugation at 2000 × g for 20 min. to remove dead cells. A third centrifugation at 10,000 × g for 30 min. was used to remove cell debris before culture medium was ultracentrifuged at 100,000 × g for 60 min. in an Optima TLX ultracentrifuge with 60 Ti rotor (Beckman Coulter). The NV-free supernatant was removed, and pellets containing NVs and proteins were resuspended in PBS. The suspension was finally ultracentrifuged for an additional 60 min. at 100,000 × g at 4°C to collect final NV pellets.

For isolation of cytoplasmic vesicle-free fraction, fHASCs treated or not with IFN-γ, were resuspended in 12% Suc, 10 mM KCl, 100 mM Tris-Cl, pH 7.8 and 2 mM MgCl_2_) and homogenized. A continuous sucrose gradient between 16% and 55% was made using the same buffer and 800 μl of the two homogenates were loaded on top of the gradient. After centrifugation at 141,000 × g for 4 hrs at 4°C in a Beckman SW28 rotor (Beckman Coulter), the first three fractions of the gradient—in which cytoplasmic vesicles free proteins are usually recovered—were mixed, and an aliquot of this sample was treated with loading buffer and then analysed by SDS-PAGE. The hydrodynamic size distribution of NVs was measured by dynamic light scattering (DLS) using a NICOMP 380 ZLS equipped with a 55 mW He-Ne Coherent Innova 70-3 Laser source at 654.0 nm and an APD detector (Particle Sizing System). Samples were analysed after centrifugation (4000 r.p.m. 3220 × g, 15 min.) in Vivaspin 6 tubes (MWCO 30 kD; Sartorius, Goettingen, Germany). DLS analyses were compared with the distribution obtained by statistical particle size analysis of scanning electron microscopy (SEM) images (630 random counts) using ImageJ software. Vesicles from HASCs were also identified after carboxyfluorescein succinimidyl ester (CFSE) staining by FACS, using flow cytometric assessment of nano-sized beads as a standard (Life Technology, Monza, Milan, Italy; Fig. S4).

For vesicle counting, fHASC-derived vesicles were incubated with CFSE for 20 min. in the dark at room temperature. After incubation, 100 μl of Flow-Count Fluorospheres (Beckman Coulter) were added to an equal volume of NV preparation for the absolute count. Analyses were performed on the EPICS XL Flow-Cytometer (Beckman Coulter). Absolute event count (events/μl) was done using the following equation: (A/B) × C, where A = total number of counted events, B = total number of Flow-Count Fluorospheres and C = nominal concentration of Flow-Count Fluorospheres. At least 1000 Flow-Count Fluorospheres were acquired to ensure adequate accuracy. A Flow Cytometry Sub-micron Particles Reference Kit (Molecular Probes by Life Technologies) was used to establish appropriate size gates.

### Scanning electron microscopy

For scanning electron microscopy (SEM) examination, untreated or INF-γ–stimulated fHASCs were grown on a cover glass for 2 days and fixed in 1.5% glutaraldehyde for 15 min. at room temperature and washed three times with PBS. Cells were dehydrated stepwise, first in ethanol and then by critical point drying 31. HASC-derived NVs were fixed in 1.5% glutaraldehyde for 15 min. at room temperature, washed with water, sedimented on glass coverslips and then allowed to dry at room temperature. SEM images were obtained using a field emission gun electron scanning microscope (LEO 1525 Zeiss; Thornwood, NY, USA) with Cr metallization using a high-resolution sputter Q150T ES-Quorum apparatus (24 sec. sputter at a current of 240 mA). Chromium thickness was ∽10 nm.

### Skin transplantation

Skin transplantation was performed according to the method described by Stubenitsky *et al*. 32. Briefly, full thickness tail skin grafts (0.5 × 0.5 cm) from donor mice (BALB/c) were grafted on the lateral flank of recipient mice (C57BL/6) under general anaesthesia in two sequential stages: induction with O_2_/NO_2_ (2:1) at 1 l/min. plus isoflurane at 5% and maintenance with O_2_/NO_2_ (2:1) at 1 l/min. plus isoflurane at 1 and 2%. The graft was protected by bandage and was removed on day 4, and mice were monitored daily from day 4 to day 20 by visual inspection. Graft rejection was defined as the complete destruction or desiccation of the grafted skin on inspection. In selected groups, C57BL/6 recipient mice were injected intraperitoneally with fHASCs (2 × 10^6^) 24 hrs before transplantation. Skin graft biopsies, taken from each recipient on the day of rejection or on day 20, were observed by removing a single tissue portion from the graft, so to maintain 2–3 mm of recipient skin (through the muscular band) and allow for visualization of the cleavage plane between donor and recipient skin. Paraffin-embedded skin sections (3 or 4 μm), obtained by cutting the samples at regular intervals of at least 200 μm, were stained with haematoxylin/eosin and examined by light microscopy.

### Statistical analysis

All *in vitro* determinations are means ± SD from three independent experiments, and were evaluated by Student’s *t*-test. All *n* values were computed by power analysis to yield a power of at least 80% with an α-level of 0.05. The log-rank test was used for paired data analyses of Kaplan–Meier graft survival curves and GraphPad Prism version 6.0 (San Diego, CA, USA) was used for all analyses and graph preparation.

## Results

### Isolation of stem cell types from amniotic fluid

We used human amniotic fluid to isolate HASCs. Cells were characterized by a fibroblast-like morphology (Fig.1A), a doubling time of 14.0 ± 0.43 hrs (Fig. S1) and are hereafter referred to as ‘fast’ human amniotic fluid stem cells, or fHASCs. fHASC morphology was fibroblast-like, and colony shape resembled dermatoglyphics (Fig.1B). Notably, fHASCs could be cultured *in vitro* for more than two hundred generations, in the absence of feeder layers. Overall, of 1500 samples examined over time, about 40% gave rise to fHASCs which could be selectively cloned into homogenous populations of fHASCs. In addition, fHASCs could be cryopreserved for prolonged periods of time, so as to allow maintenance of their proliferative properties. The data in this study were generated using five different fHASC lines, three with a female and two with a male karyotype.

### fHASCs exhibit stem cell-like profiles and express key markers of pluripotency

fHASCs expressed the main markers typical of stromal stem cells 33, as they stained positive for the major histocompatibility antigens (HLA-ABC), CD10 (Common Acute Lymphoblastic Leukemia Antigen), CD29 (integrin β1 chain), CD44 (hyaluronan receptor), CD73 (ecto-5′-nucleotidase), CD90 (Thy-1 membrane glycoprotein) and CD105 (endoglin), but they were negative for class II major histocompatibility antigens HLA DR and haematopoietic markers, including CD11b, CD14, CD34, CD38, and CD49d (Fig. S2).

**Figure 2:**
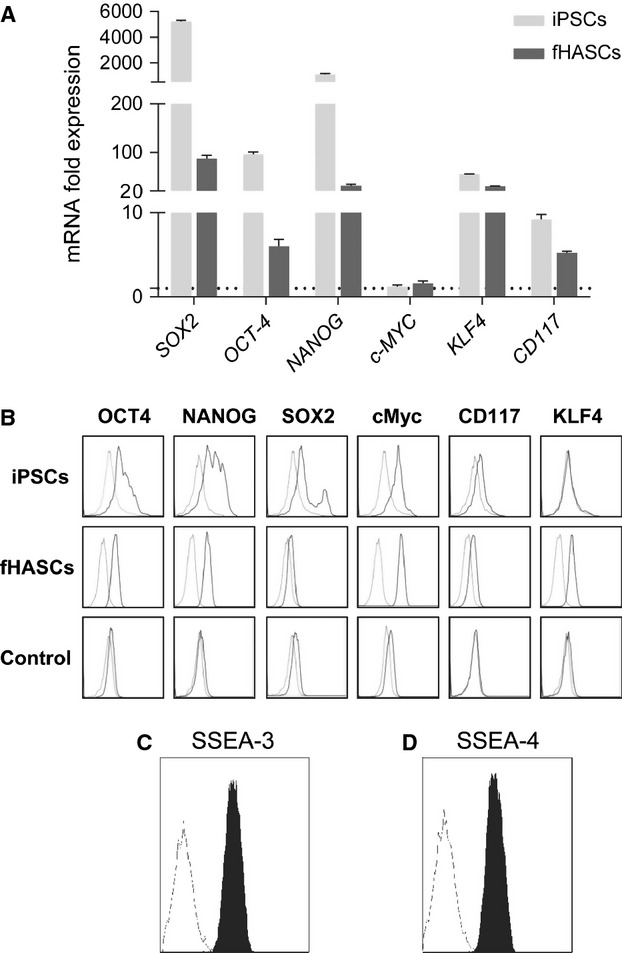
Stem cell-like phenotype of fHASCs. (A) Analysis of expression of pluripotent markers by real-time PCR in fHASCs. Data (mean ± SD of three experiments. This is explained a few lines below, prior to (B)) are presented as normalized transcript expression in the samples relative to normalized transcript expression in control cultures (that is, a pool of eight aliquots of amniotic fluid cells, in which fold change = 1; dotted line). iPSCs, derived as described in Material and methods, were used as a positive control. *P < 0.05; **P < 0.001 (Shapiro test). (B) Analysis of pluripotent marker expression by FACS in fHASCs. (C and D) Analysis of the embryonic stem cell markers SSEA-3 and SSEA-4 by flow cytometry in fHASCs. Data are representative of three independent experiments conducted on five fHASC lines.

We evaluated the expression of the main transcription factors associated with the maintenance of an undifferentiated state and with pluripotency in ESCs 34–36, including *OCT-4A* (POU class 5 homeobox, Octamer-binding protein 4), NANOG, Sex-determining region Y (SRY)-box 2 (*SOX2*), *c-MYC,* and *KLF4* (gut-associated Kruppel-Like Factor 4; Fig.2A). All these pluripotency markers were expressed by the fHASCs at the mRNA level (Fig.2A and B). Under the same conditions, mRNA from pooled amniotic fluid cells were negative for the expression of those markers, which were instead expressed by a positive human iPSC culture. Cytofluorimetric analysis confirmed that fHASCs expressed the protein markers of pluripotency to the same extent as iPSCs, except for SOX2 (Fig.2A). As shown by the mRNA data, no protein expression for the same markers was detected in amniocytes (Fig.2A) Interestingly, KLF4 was expressed in fHASCs and iPSCs at the mRNA level (Fig.2A), but only fHASCs expressed the relevant protein (Fig.2B). We found that fHASCs exquisitely expressed CD117, both at the mRNA and protein levels (Fig.2A and B). In all fHASC lines, CD117 could be detected intracellularly but not on the cell surface.

To determine whether fHASCs expressed other embryonic markers—generally unexpressed by ASCs—we evaluated the presence of SSEA-3 and SSEA-4. fHASC lines were characterized by high levels of SSEA-3, and SSEA-4 (Fig.2C and D). Cryopreservation of fHASCs affected none of those markers, their expression remaining unaltered for longer than 1 year, with preserved viability and self-renewal capacity, and cells manifested biological activity at only 5 hrs of thawing. Moreover, the expression of mesenchymal, stemness and pluripotency markers did not undergo changes. No chromosomal alterations were detected. The same results were obtained in repeatedly frozen and thawed cells.

### Clonogenic capacity and EB formation in HASCs

fHASCs were assayed for their capacity to be cloned by serial dilution *in vitro*. fHASCs would expand from a single cell in the absence of any feeder layers (data not shown). fHASC clones maintained their proliferative capacity to an extent comparable to the parental fHASC line (Fig. S3). All five fHASC lines routinely showed 100% clonogenic efficiency. All of the tested clones (*n* = 5) maintained the same expression of all surface and intracellular markers as the parental line, except for CD105, which was down-regulated in all clones.

**Figure 3:**
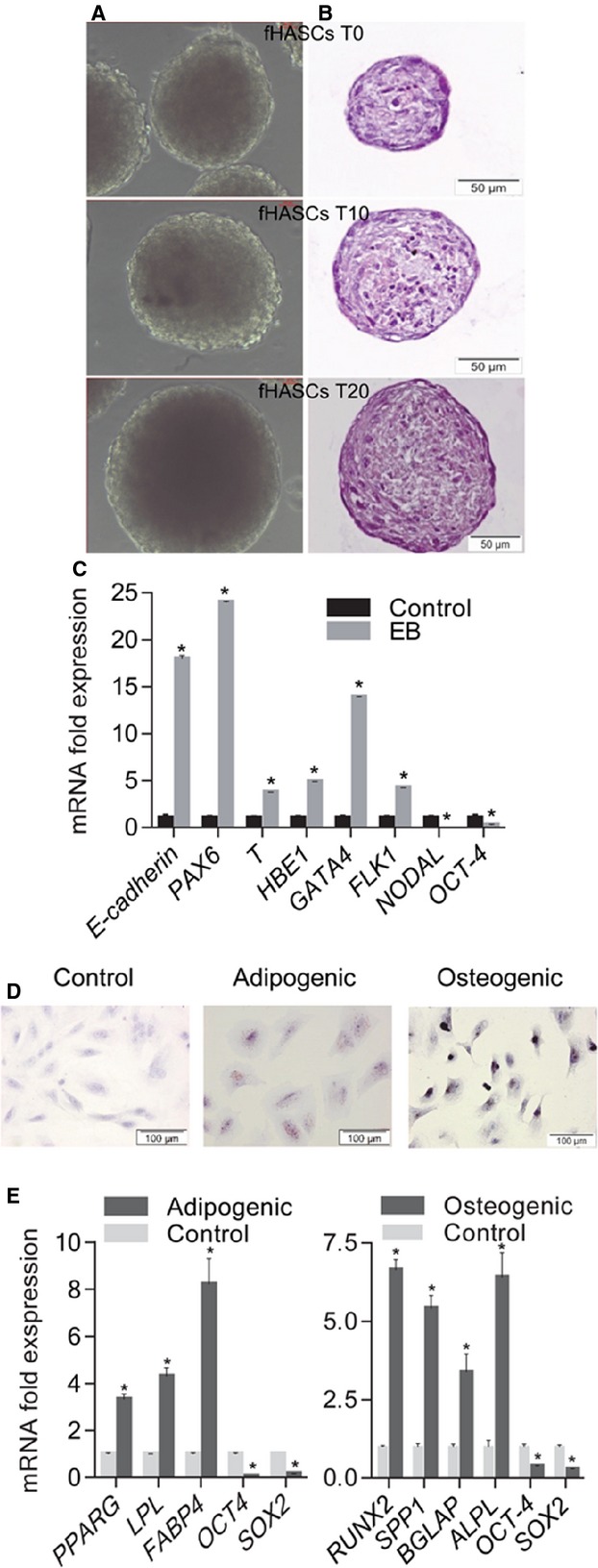
Differentiation potential HASCs. (A) Representative pictures of embryoid bodies (EBs) formed by HASCs in hanging-drop cultures. fHASC-derived EBs that were capable of sustained growth *in vitro* (T0, 4 days; T10, 10 days; T20, 20 days). (B) EBs stained with haematoxylin/eosin. EB images were obtained by phase-contrast microscopy (×20 magnification). (C) Marker expression before and after EB formation as assessed by RT-PCR (normalized to β-actin). (D) Representative pictures of fHASC differentiation into adipocytes and osteocytes respectively. Differentiation was monitored with Sudan III staining of lipid droplets in terminal adipocyte differentiation and von Kossa staining of calcium deposition in the extracellular matrix in terminal osteoblast differentiation; nuclear staining was with Mayer’s haematoxylin. (E) qRT-PCR analysis of mRNA levels of specific adipogenic (PPARG, LPL, FABP4) and osteogenic (ALPL, OCN, OPN, RUNX2) markers for fHASCs). Analysis was performed at day 21 of differentiation as compared to day 0, which corresponds to fold change = 1. All data shown are representative of three independent experiments conducted on five fHASC lines (*P < 0.05).

To analyse the potency of HASCs, we investigated whether fHASCs would form teratomas *in vivo*, and were capable of generating EBs and differentiating into mesodermal lineage cells, adipocytes or osteocytes *in vitro*. The teratoma assay and EB formation are used as an *in vivo* means of testing pluripotency. The teratoma assay is not only a pluripotency assay but it also unravels any tumourigenic potential. Notably, fHASCs did not originate teratomas when injected into immunosuppressed mice (data not shown), but they were able to form EBs 13. The incidence of EB formation (expressed as percentage of attached EBs recovered from the fifty hanging drops being seeded) was investigated in five independent experiments. Under these conditions, fHASCs showed an overall 80% EB-forming ability (Fig.3A and B). By RT-PCR analysis, we obtained evidence that the EBs from fHASCs expressed markers specific of the three germ layers, namely, E-cadherin (epithelial), PAX6 (ectodermal), T (BRACHYURY) and HBE1 (mesodermal), and GATA4 and FLK1 (endothelial). This was associated with decreased NODAL and OCT-4 expressions (Fig.3C). When fHASCs (Fig.3D) were cultured under adipogenic or osteogenic conditions, they exhibited intense cytoplasmic staining with Sudan III (showing accumulation of lipid vacuoles) or were positive on von Kossa staining, respectively. These data were confirmed by expression analysis of adipogenic or osteogenic differentiation markers (Fig.3E). Overall, these data suggested that fHASCs represent pluripotent stem cells with characteristics common to ESCs and MSCs. To further assess any immunoactive properties, we used five different clones, each derived from a single fHASC line.

### fHASCs up-regulate IDO1 after treatment with IFN-γ

**Figure 4:**
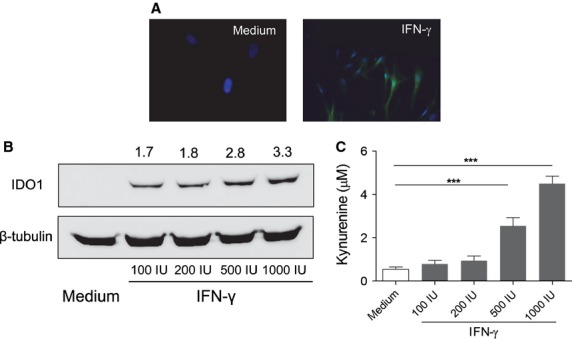
fHASCs express immunoregulatory IDO1. (A) IDO1 detection by immunofluorescence staining in fHASCs that were treated or not with 1000 U/ml IFN-γ for 24 hrs. Nuclei were counterstained with DAPI (blue) and IDO1 was revealed using a fluorescently labelled secondary antibody. Data are representative of one of three independent experiments using five fHASC lines. (B) Western blot analysis of IDO1 protein expression in fHASCs treated or not with 100, 200, 500 or 1000 U/ml IFN-γ for 24 hrs; β-tubulin was used as a control. (C) IDO1 enzymatic activity was measured by HPLC quantification of tryptophan conversion into kynurenine. Data are representative of three independent experiments involving five fHASC lines ***P<0.05–0.01.

IDO1 is an immunoregulatory enzyme that fosters the generation and/or activation of IL-10– and TGF-β–producing regulatory T (Treg) cells 25–27. We investigated IDO1 expression in fHASCs and the possible contribution of IDO1 to immunoregulation by fHASCs. fHASCs did not express IDO1 protein under basal conditions, but they did so after treatment with IFN-γ (Fig.4A). Specifically, 24-hr treatment with increasing concentrations of human IFN-γ promoted a dose-dependent induction of IDO1 (Fig.4B). Notably, and consistent with IDO1 protein expression, IFN-γ–treated fHASCs, but not unstimulated fHASCs, were capable of dose-dependently converting tryptophan to kynurenine, as detected by HPLC analysis (Fig.4C). Under these conditions, IDO2 (a paralogue of IDO1) was not inducible in fHASCs.

### IDO1 induction in fHASCs is instrumental for their immunoregulatory function, requiring no cell-to-cell contact

**Figure 5:**
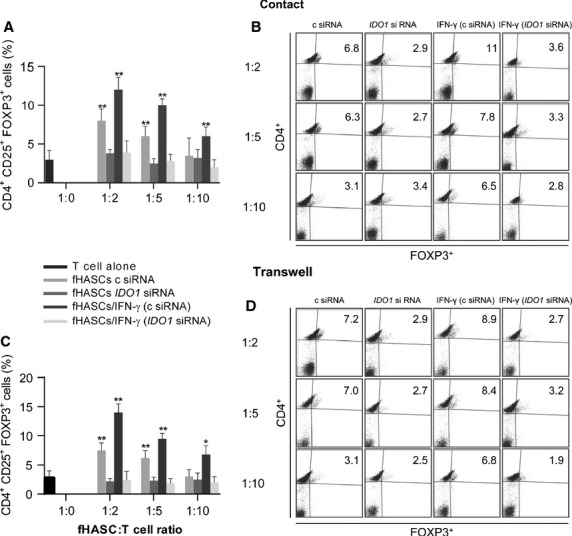
Treg induction by fHASCs requires IDO1 but not cell–cell contact. (A) Regulatory T-cell enumeration by cytofluorimetric analysis, presented as the proportion of CD4^+^ CD25^+^ FOXP3^+^ cells in PBMCs after 96-hr coculture with fHASCs that had been treated or not with IFN-γ, or transfected with *IDO1* siRNA (*IDO1* siRNA) or control siRNA (c siRNA). (B) Representative plots from one of three experiments. (C and D) Panels refer to the same experimental conditions as in (A) and (B) but with the cocultured PBMCs and fHASCs being separated by transwell inserts. Data shown in A and C are representative of three independent experiments involving five fHASC lines (**P < 0.01; *P < 0.05).

To investigate whether the IFN-γ–induced expression of IDO1 in fHASCs has biological relevance *in vitro*, we explored the effects of IFN-γ conditioning on cocultures of fHASCs with naïve PBMCs at specific fHASC-to-PBMC ratios. We assessed the ability of IFN-γ–stimulated fHASCs to induce Treg cells, using unstimulated fHASCs as a control. We transfected groups of fHASCs with small interfering RNA (siRNA) targeting *IDO1* (or with control siRNA) before treating cells with IFN-γ. The addition of IFN-γ-stimulated fHASCs to PBMCs—stimulated, in turn, with anti-CD3/CD28—significantly inhibited proliferation (data not shown). To assess whether the proliferating cells expressed specific Treg-cell markers, we analysed the effect of fHASC clones on the expansion of CD4^+^ CD25^+^ FOXP3^+^ Treg cells, under these culture conditions and over time. At 96 hrs, the proportion of Treg cells was significantly increased in the CD4^+^ T-cell population by the addition of fHASC clones, which dose-dependently induced a higher frequency of FOXP3^+^ CD25^+^ (Treg) cells in the CD4^+^ cell population, contingent on IDO1 expression by fHASCs (Fig.5A and B). Unstimulated fHASCs promoted Treg-cell differentiation, again requiring IDO1 expression in the fHASCs (Fig.5A and B). Consistent with these findings, we found high levels of IFN-γ in coculture supernatants, and analysis of IDO1 protein expression in fHASCs revealed IDO1 induction after fHASC coculture with PBMCs.

To determine whether cell-to-cell contact was required for immunoregulatory effects, we established HASC-PBMC cocultures using the transwell system. fHASCs were cultured in the lower chamber and activated as above. Notably, Treg-cell induction by fHASCs was unchanged when PBMCs were physically separated by the transwell membrane, and the effect required IDO1 expression by fHASCs (Fig.5C and D). Collectively, these results suggested that HASCs mediate their immunoregulatory effects in a paracrine fashion, which ultimately requires IDO1 in the HASCs.

### fHASCs release IDO1-containing vesicles that promote immunoregulatory functions

**Figure 6:**
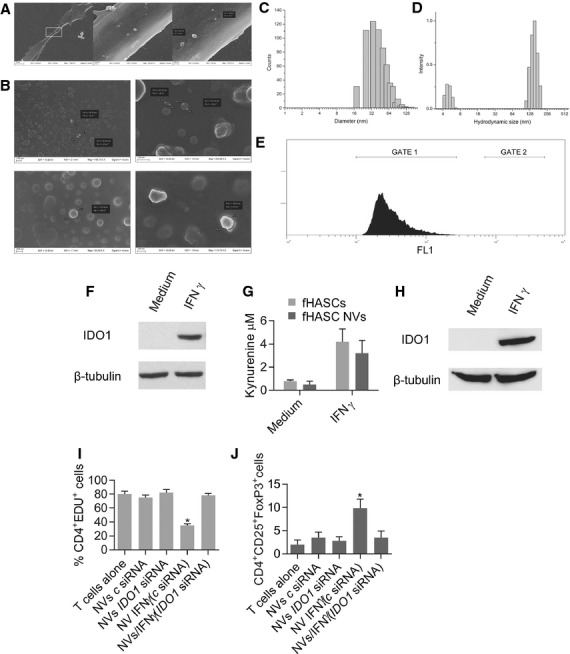
IFN-γ-stimulated fHASCs release nanovesicles that contain active IDO1 protein. (A) SEM images of fHASCs captured at different magnifications and showing vesicle budding from the plasma membrane. Nanovesicles (NVs) were purified from fHASCs, treated or not with IFN-γ and were visualized by SEM (B). (C) Size distribution of NVs obtained by statistical analysis of SEM images (630 random counts) manipulated using ImageJ software. (D) Hydrodynamic size distribution by DLS of NVs purified from fHASCs. (E) FACS analysis of CFSE-labelled fHASC-derived vesicles. One representative experiment of three. (F) NVs were purified from fHASCs, treated or not with IFN-γ and were subjected to lysis and analysis by Western blot to determine protein content. (G) Kynurenine production by fHASC-derived NVs or intact fHASCs that had been pre-treated with IFN-γ for 24 hrs prior to analysis. (H) Cytoplasmic vesicle-free fraction (CVF) was purified from fHASCs, treated or not with IFN-γ and was analysed by Western blot for IDO1 protein content. One representative experiment of three. (I) Cell proliferation was measured by cytofluorimetric analysis of CD4^+^ EdU^+^ cells in PBMCs after coculture with either fHASC-derived NVs or intact fHASCs that had been treated with IFN-γ for 24 hrs and/or transfected with *IDO1* siRNA (*IDO1* siRNA) or control siRNA (c siRNA). (J) Regulatory T-cell enumeration by cytofluorimetric analysis of CD4^+^ CD25^+^ Foxp3^+^ cells after 96-hr coculture. Shown are mean values ± SD from three independent experiments involving five fHASC lines (*P < 0.05).

Stem cells of different origin produce vesicles which are instrumental in their paracrine communication 37,38. Because the immunoregulatory properties of fHASCs were not dependent on cell-to-cell contact, we investigated the ability of fHASCs to release vesicles that might be responsible for their regulatory effects. We found that both unstimulated and IFN-γ–stimulated fHASCs would produce vesicles, 40–200 nm in size, when analysed by SEM (Fig.6A–C). A deeper characterization of vesicle size distribution provided distinct population ranges when size was measured by statistical analysis of SEM images (Fig.6C) or by DLS (Fig.6D). In particular, image analysis revealed a population 20–120 nm in size (Fig.6C), and DLS indicated a size of 100–250 nm (Fig.6D). This suggested partial aggregation of the vesicles when dispersed in the medium. This behaviour is not unusual, as fine particles or vesicles of a few tens of nm tend to cluster as a result of the predominant influence of van der Waals forces. Moreover, cytofluorimetric analysis of vesicles by means of sub-micron particle size reference beads (Fig. S4) showed that fHASCs did not produce microvesicles but only NVs under these culture conditions (Fig.6E).

On analysing the immunoregulatory functions of these vesicles we found that purified vesicles from IFN-γ–stimulated fHASCs contained functional IDO1 protein, as detected by immunoblot analysis (Fig.6F) and were capable of kynurenine production (Fig.6G). IDO1 was also expressed in vesicle-free lysates from IFN-γ–stimulated fHASCs (Fig.6H), suggesting that IDO1 may have different intracellular localizations. Because IDO1 induction in fHASCs was responsible for their immune regulatory effects on PBMCs, we explored the ability of purified fHASC-derived vesicles to exert regulatory function. Human PBMCs were cultured with vesicles purified from one million fHASCs (∽6–8 × 10^6^ NVs), either untreated or treated with IFN-γ for 48 hrs, to induce the IDO1 protein. Much like whole cells, the IDO1-expressing vesicles were capable of both restraining T-cell proliferation and promoting Treg-cell expansion in PBMCs. *IDO1*-specific silencing in donor HASCs completely abrogated these functions (Fig.6I and J). These data may be among the first to demonstrate that fHASC-derived vesicles recapitulate the immunoregulatory functions of HASCs, and that those effects can be traced to IDO1 presence in the vesicles.

### fHASCs promote allograft survival in association with Treg cell expansion *in vivo*

**Figure 7:**
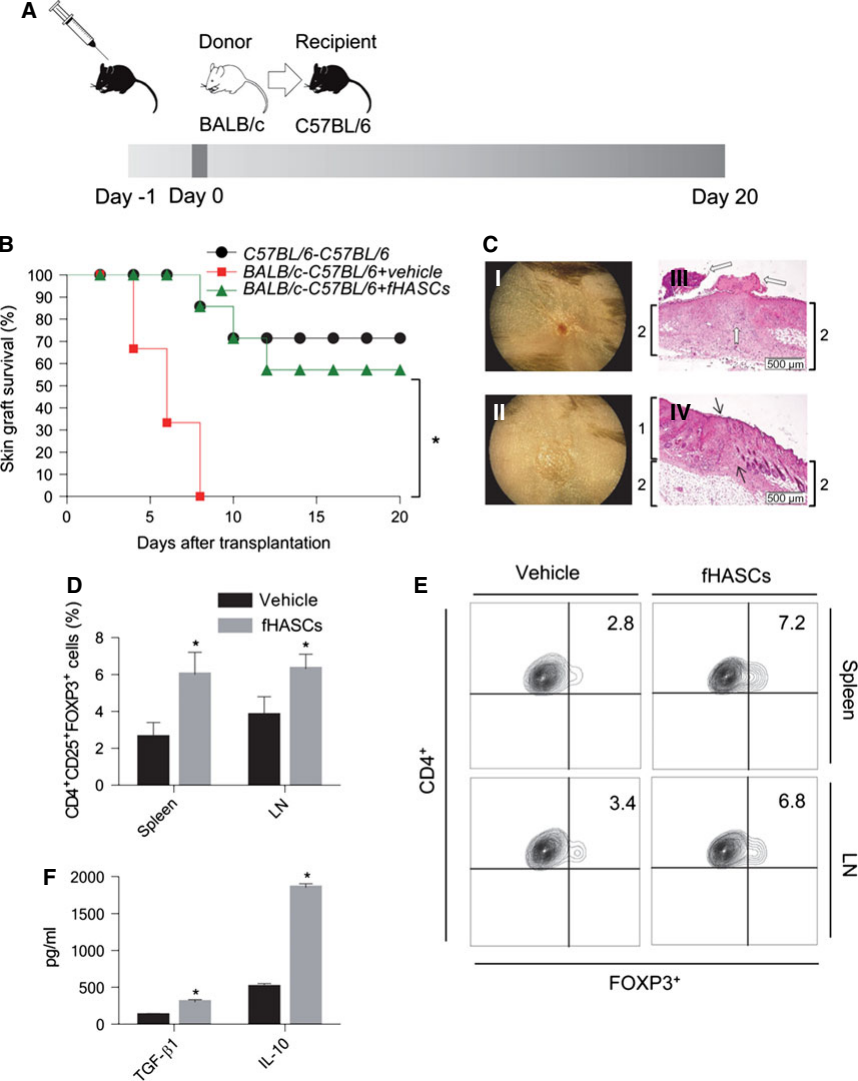
fHASCs promote skin allograft engraftment. (A) Experimental protocol for fHASC-dependent skin allograft tolerance induction. BALB/c tail skin grafts were used as donor material while C57BL/6 were transplant recipients. For tolerance induction, C57BL/6 mice were treated intraperitoneally with a single dose of fHASCs (2 × 10^6^ cells) on day −1. Skin transplantation was performed on day 0. (B) Post-transplantation survival curves in syngeneic skin graft only (C57BL/6 /C57BL/6), black line (*n* = 10); in skin allograft only (BALB/c /C57BL/6/vehicle), red line (*n* = 10); and in skin allograft with fHASC treatment (BALB/c /C57BL/6/fHASCs), green line (*n* = 10). Observation period, 20 days. Log-rank test (*P < 0.05). (C) Representative skin graft appearance and histological evaluation. (I) Complete graft rejection with the scar from vehicle-treated mice; (II) completely healed skin graft without evidence of rejection from fHASC-treated recipients. Haematoxylin and eosin staining of skin graft sections from vehicle (III) or fHASC (IV) recipients. (D) Frequency of CD4^+^ CD25^+^ Foxp3^+^ Treg cells in spleens and graft-draining lymph nodes from treated recipients. Data are mean values ± SD of three independent experiments (*n* = 9). (E) Representative plots from one of three experiments. (F) TGF-β and IL-10 production from purified CD4^+^ cells from graft-draining lymph nodes, in treated recipients. Data are mean values ± SD of three independent experiments (*n* = 9).

To investigate the *in vivo* immunomodulatory effects of fHASCs, we used a skin allograft model of BALB/c skin into C57BL/6 recipient mice (Fig.7A). The results are shown in Figure7B and C. Treatment with fHASCs prolonged skin graft survival in allogeneic recipients as compared to vehicle-treated controls. Histological analysis revealed a dense lymphocyte infiltrate, scab formation and a rapid loss of volume in the control group. By contrast, no sign of inflammation could be observed in the fHASC-treated groups, where there occurred obvious signs of re-epithelization in the epidermis (Fig.7C). Notably, the extent of complete skin allograft engraftment in mice treated with fHASCs was similar to that observed in a syngeneic setting (60% for allogeneic *versus* 70% for syngeneic recipients). Consistent with what observed *in vitro*, we found that fHASC administration increased the frequency of CD4^+^ CD25^+^ Foxp3^+^ Treg cells both in the spleens and in graft-draining lymph nodes of allogeneic recipients (Fig.7D and E). Moreover, CD4^+^ T cells purified from the fHASC-treated mice produced higher levels of two Treg-associated cytokines, IL-10 and TGF-β1 (Fig.7F). Overall, these results demonstrate that administration of fHASCs will prevent allograft rejection in a skin transplantation model and that this effect is associated with fHASC-induced Treg responses *in vivo*.

## Discussion

Stem cells in general, and those from amniotic fluid as well, are highly heterogeneous, and each population may be endowed with specific immunoregulatory properties. Although it is widely documented that MSCs have potent immunomodulatory functions *in vitro* and *in vivo* 39, it has remained unclear whether additional stem-cell types manifest the same or similar potential. By a method not requiring prior immunoselection, we isolated stem cells from human amniotic fluid, routinely drawn from pregnant women at 16–17 weeks of gestation for diagnostic purposes. After one round of cell culture, stem cell isolation consisted of selecting cultures containing cells with a prevalent fibroblast-like morphology and a colony shape similar to dermatoglyphics. fHASCs expressed the main stromal markers but also transcription factors associated with the maintenance of an undifferentiated state and pluripotency in ESCs. Moreover, they were able to differentiate into adipocytes and osteocytes. Moreover, fHASCs manifested sustained self-renewal capacity and could be cloned and propagated for >200 generations *in vitro*, as they retained phenotypic and chromosomal stability (data not shown), thus allowing for detailed characterization at the clonal level. Freezing and thawing of fHASC lines affected neither their phenotype nor biological activity.

Several *in vitro* protocols have been described in the literature to test the differentiation potential of stem cells, including (*i*) measurements of pluripotency markers, *e.g*. OCT-4, NANOG, SOX2 40, as well as of glycolipid antigens, such as SSEA3 and SSEA4; (*ii*) EB formation and (*iii*) differentiation into specific lineages 41. The teratoma assay is used as an *in vivo* means of testing pluripotency of the cells, namely, the ability of those cells to generate cells/tissues of all three germ layers. The teratoma assay is not only a pluripotency assay but it is also unravels any tumourigenic potential, and it is of major importance to address safety issues of new stem cell-based therapies. We used these tests to explore the differentiation capacity of our fHASCs. Notably, fHASCs have all the characteristics of pluripotent cells, but they do not generate teratomas. These results are in agreement with those by De Coppi *et al*. 6, showing that amniotic fluid pluripotent stem cells would not generate teratomas. Overall, these data suggest that fHASCs represent pluripotent stem cells with characteristics common to ESCs and MSCs 9,10. Moreover, fHASCs show some peculiar characteristics. Their duplication time (14 hrs; regardless of medium or culture conditions) is significantly lower than that of MSCs or AFSCs from amniotic liquid (32 hrs); also, unlike AFSCs or AFMSCs 6, they do not express CD117 intracellularly. As reported for MSCs, we found that fHASC clones exhibited potent immunomodulatory properties, upon treatment with IFN-γ, which would confer strong IDO1 competence on fHASCs and their clones. IDO1 can be induced in several immune cells 26 and it is up-regulated in stem cells by inflammatory stimuli 42. In our setting, IDO1 induction in fHASCs was required for the cells to manifest immune regulatory function. IDO1 is, indeed, pivotal in mammalian pregnancy, protecting the foetus from the fetomaternal immunological conflict 22. Yet, IDO1 and Treg cells may have coevolved in mammals to broaden their function well beyond their initial task of protecting the foetus, such that, in acquired immunity, IDO1—with its dual enzymic and signalling function—has turned into an important component of the peripheral generation and effector function of Treg cells 25.

We found that coculturing IFN-γ–treated and IDO-expressing fHASCs with allogeneic PBMCs promoted a significant decrease in T-cell proliferation and an increase in the Treg-cell component. The effect did not require cell-to-cell contact between PBMCs and fHASCs. The mechanisms underlying MSC immunosuppressive effects and other stromal cells are typically mediated by direct cell-to-cell interactions in combination with soluble factors 42. These immunosuppressive effects include suppression of T-cell proliferation 42. In our setting, both the antiproliferative effects and the induction of Treg cells could be mimicked by coculturing PBMCs with NVs purified from the IFN-γ–treated fHASCs. These NVs contained functionally active IDO1 protein. To the best of our knowledge, this is the first report to describe the presence of functionally active IDO1 in NVs released from human stem cells.

Vesicles released by different cell types are recognized as being highly relevant cell communication 43. Exosomes derived from dendritic cells genetically engineered to express IDO1 abundantly express the IDO1 protein and manifest therapeutic activity, immunological in nature, in a collagen-induced arthritis model 44. IDO1-expressing exosomes may act to deliver IDO1 to dendritic cells, T cells, or other tissue cell types such as macrophages. This would render recipient cells tolerogenic, *via* mechanisms including kynurenine-dependent fostering of Treg-cell differentiation 45. No kynurenine production, however, was detected in Exo/IDO1 delivered by dendritic cells 44. In contrast, we found that NVs released by fHASCs contained functional IDO1 protein (Fig.5B).

The multitude of soluble inhibitory factors produced by MSCs suggests that the cellular and molecular composition of the environment—in which stem cells make contact with T or other cells—is of fundamental importance in promoting either anti-proliferative or prosurvival effects. This might apply to other stem cell types as well, particularly those derived from amniotic fluid. Further studies might clarify whether other inflammatory stimuli, particularly those from pathogen-derived ligands, promote similar or distinct immunoregulatory functions. Interestingly, a recent report showed that poly (I:C), a Toll-Like Receptor 3 ligand, improves the immunosuppressive abilities of MSCs, and this may provide an opportunity for potentially novel therapies in sepsis 46. While purifying exosomes from dendritic cells might be a difficult task, we found that high numbers of NVs containing IDO1 protein could be easily obtained from *in vitro* cultured fHASCs treated with IFN-γ. IDO1 may be more stable when present in NVs that protect the protein from degradation. Moreover, IDO1-containing vesicles may be better suited for directing IDO1 protein to specific target cells.

Allograft rejection remains an obstacle for successful organ transplantation. Although different types of immunosuppressive agents are effective for controlling rejection and prolonging graft survival, drug treatment is of limited utility because of side effects and toxicity. Therefore, it is necessary and urgent to identify new candidates for inducing allotolerance. Recently, it has been reported that stem cells induce the immunosuppressive activity of Treg cells *in vitro* 43. Here, we demonstrate that fHASCs do promote allograft acceptance in a model of skin graft and this is associated with an increase in the Treg population. In transplantation, Treg cells are involved in tolerance induction. Therefore, it is possible fHASCs, by virtue of IDO1 expression, act *in vivo* to increase the frequency of Treg cells and their soluble products.

Finally, this study highlights the potential therapeutic use of fHASC lines and of purified NVs from HASCs. The use of NVs instead of whole cells allows for more stability with no loss of activity. While this study focuses on the immune regulatory action of IDO-containing NVs derived from HASCs in an entirely *in vitro* setting, NVs may also have regulatory effects under physiopathologic *in vivo* conditions, where IDO1 is known to exert protective or regulatory effects 22–24.

## Supporting Information

**Figure S1** HASCs growth curve *in vitro*.

**Figure S2** Phenotypic analysis of HASCs.

**Figure S3** Growth rates of the parental fHASC line and fHASC clones.

**Figure S4** Size distribution of reference beads by FACS.

**Table S1** Real-time PCR primer sequences.
