# Supplementary material for: Stem cells from human amniotic fluid exert immunoregulatory function via secreted indoleamine 2,3-dioxygenase1
Source: J Cell Mol Med. 2015 Mar 17;19(7):1593–605. doi: 10.1111/jcmm.12534 (PMC4511357; doi:10.1111/jcmm.12534)
Supplement: Supplementary file 1 [file jcmm0019-1593-sd1.doc]

**
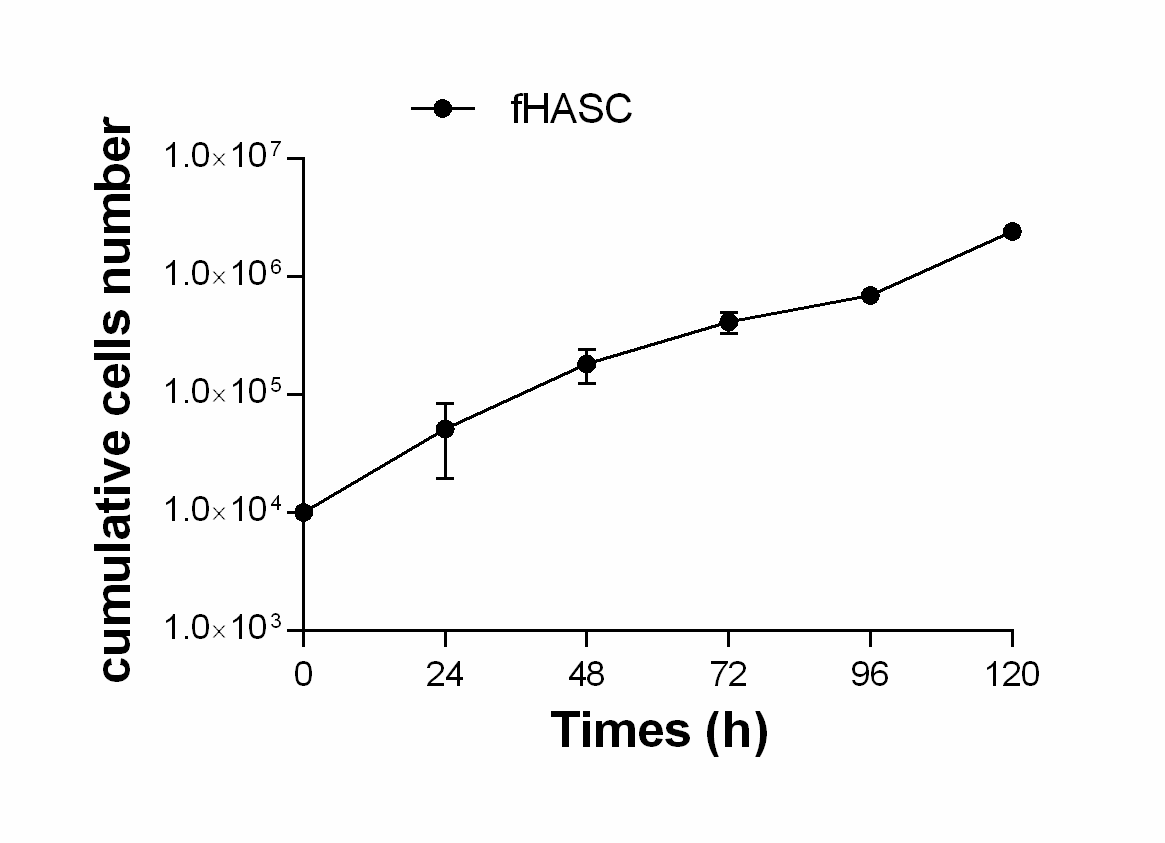
**

**Figure S1.** **HASCs growth curve *in vitro***. HASCs at fifth passage were seeded (10^4^ cells/well), and counted daily for five days. Shown are mean values ± SD of three independent samples from five different lines belonging to each population.
