# Supplementary material for: Stem cells from human amniotic fluid exert immunoregulatory function via secreted indoleamine 2,3-dioxygenase1
Source: J Cell Mol Med. 2015 Mar 17;19(7):1593–605. doi: 10.1111/jcmm.12534 (PMC4511357; doi:10.1111/jcmm.12534)
Supplement: Supplementary file 2 [file jcmm0019-1593-sd2.doc]

**
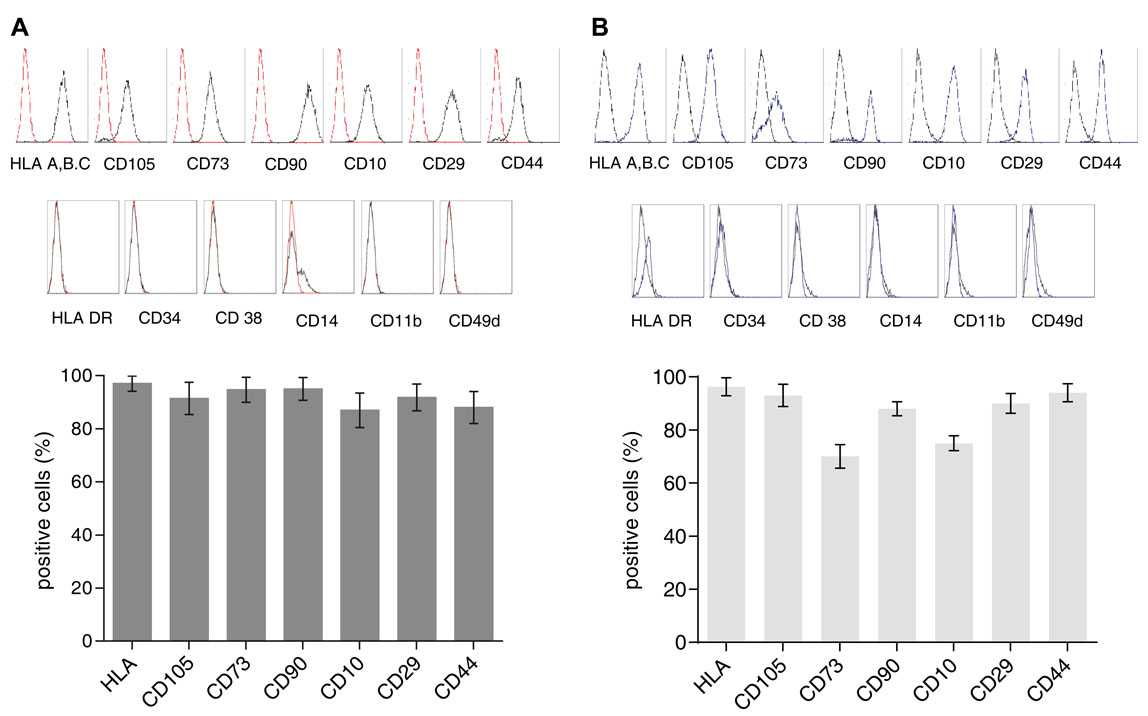
**

**Figure S2.** **Phenotypic analysis of HASCs.** Representative flow-cytometric analysis of cultured fHASCs . Red histograms indicate staining with an isotype-matched mouse IgG control antibody respectively for fHASCs.
