# Supplementary material for: Stem cells from human amniotic fluid exert immunoregulatory function via secreted indoleamine 2,3-dioxygenase1
Source: J Cell Mol Med. 2015 Mar 17;19(7):1593–605. doi: 10.1111/jcmm.12534 (PMC4511357; doi:10.1111/jcmm.12534)
Supplement: Supplementary file 3 [file jcmm0019-1593-sd3.doc]

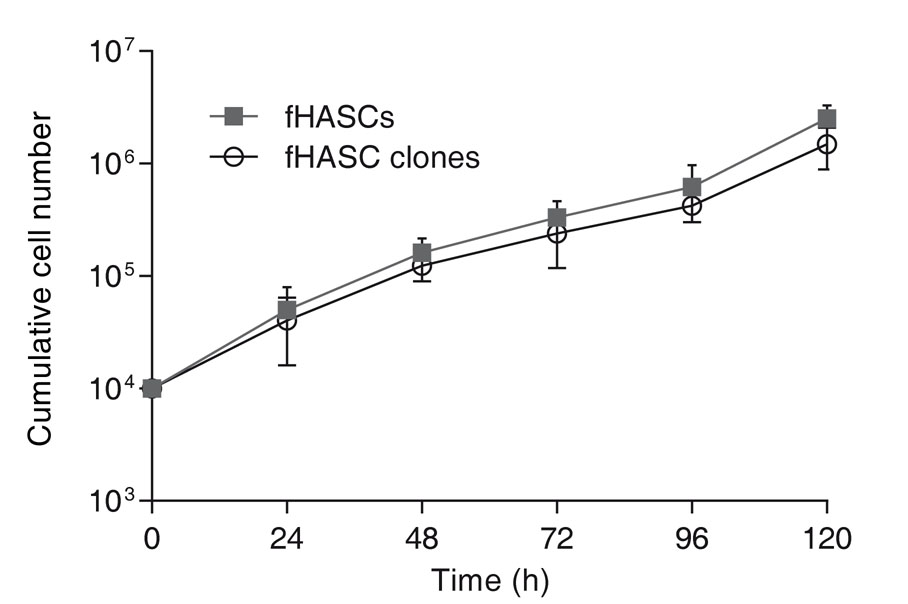


**Figure S3.** **Growth rates of the parental fHASC line and fHASC clones.**
fHASCs were trypsinized (parental cells at  passage 6, clones at passage 5) and triplicate cultures were plated out in MSCGM at matching densities of 10^4^ cells/well in a 6-well plate. The cells were subsequently re-trypsinized and counted every 24 h using Trypan Blue exclusion (Sigma-Aldrich). Population doubling time (TD) for the fHASCs and their clones was calculated using the formula: TD = t × log_2_/log(Nt/N0), where N0 = initial cell number, and Nt = number of cells harvested at time t. Three independent experiments were performed, each including three replicates. Shown are mean values ± SD of three independent samples taken from five distinct cell lines belonging to either cell type.
