# Supplementary material for: Stem cells from human amniotic fluid exert immunoregulatory function via secreted indoleamine 2,3-dioxygenase1
Source: J Cell Mol Med. 2015 Mar 17;19(7):1593–605. doi: 10.1111/jcmm.12534 (PMC4511357; doi:10.1111/jcmm.12534)
Supplement: Supplementary file 4 [file jcmm0019-1593-sd4.doc]

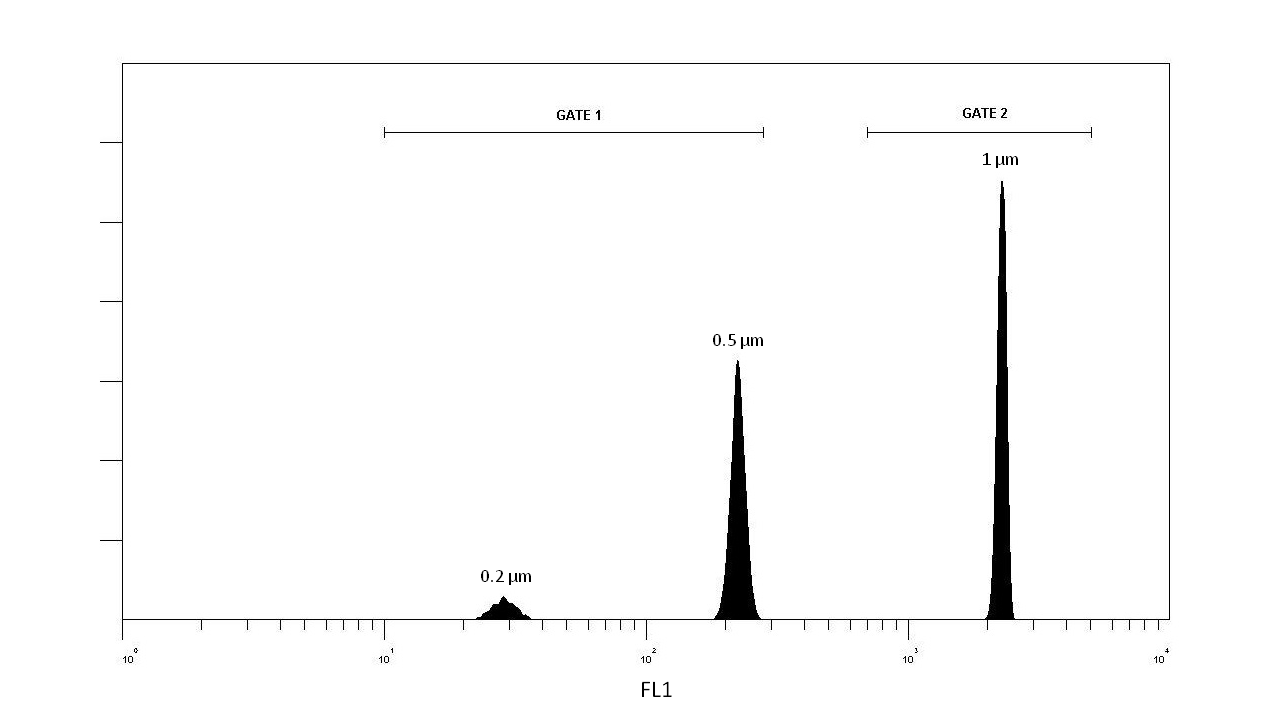


**Figure S4.** **Size distribution of reference beads by FACS.** Three components of the Flow Cytometry Sub-micron Particle Size Reference Kit (0.2-0.5-1µm), were analyzed by FACS.

Gate 1 marks microspheres with range between 0.2-0.5 µm, while gate 2 microspheres with range ≥ 0.7 µm are as marked.
