# Supplementary material for: Stem cells from human amniotic fluid exert immunoregulatory function via secreted indoleamine 2,3-dioxygenase1
Source: J Cell Mol Med. 2015 Mar 17;19(7):1593–605. doi: 10.1111/jcmm.12534 (PMC4511357; doi:10.1111/jcmm.12534)
Supplement: Supplementary file 5 [file jcmm0019-1593-sd5.doc]

**Table 1S. Real-time PCR primer sequences**

| ***Name*** | ***Accession number*** | **Forward sequences** | **Reverse sequences** |
| --- | --- | --- | --- |
| ***OCT-4*** | **NM_002701** | 5’GGGTTGAGTAGTCCCTTCGC3’ | 5’TAGCCAGGTCCGAGGATCAA3’ |
| ***NANOG*** | **NM_024865** | 5’CCACCAGTCCCAAAGGCAAAC3’ | 5’GAGGTTCAGGATGTTGGAGAGTTC3’ |
| ***SOX2*** | **NM_003106** | 5’AAGTAGTTTGCTGCCTCTTTAAG3’ | 5’GCTTCCCTCCTCCTCTGG3’ |
| ***FGF-4*** | **NM_002007** | 5’GGCGTGGTGAGCATCTTC3’ | 5’GTAGGCGTTGTAGTTGTTGG3’ |
| ***c-MYC*** | **NM_002467** | 5’GCGTCCTGGGAAGGGAGATCCGGAGC3’ | 5’TTGAGGGGCATCGTCGCGGGAGGCTG3’ |
| ***KLF4*** | **NM_004235** | 5’ACGGCTGTGGATGGAAATTC3’ | 5’ATGTGTAAGGCGAGGTGGTC3’ |
| ***NODAL*** | **NM_018055** | 5’ACATCACTTGCCAGACAGAAG3’ | 5’CCAACAGGATTAGGACACTCG3’ |
| ***IDO1*** | **NM_002164** | 5’CGGTCTGGTGTATGAAGGGTTCTG3’ | 5’AACTGAGCAGCATGTCCTCCAC3’ |
| ***E-Cadherin*** | **NM_004361** | 5’GGAGTGGGCTGTGATTGGAG3’ | 5’GGCAGACTGGAGGAACCG3’ |
| ***PAX6*** | **NM_000280** | 5’GTGGTCTTCAAGCAACAACAGCAG3’ | 5’GGGCTCTGAAATCTCGGATGTCTG3’ |
| ***T*** | **NM_003181** | 5’ATCCTGGGTGTGCGTAAC3’ | 5’CCGATGCCTCAACTCTCC3’ |
| ***HBE1*** | **NM_005330** | 5’TGCATGTGGATCCTGAGAAC3’ | 5’CGACAGCAGACACCAGCTT3’ |
| ***GATA4*** | **NM_002052** | 5’CTCCTCTGCCTGGTAATGACTC3’ | 5’AGTGTGCTCGTGCTGAAGG3’ |
| ***FLK1*** | **NM_002253** | 5’ATATCTGTCCTGATGTGATATGTC3’ | 5’CATAGCATGTCTTATAGTCATTGTTC3’ |
| ***SPP1*** | **NM_001251830** | 5’GCAGGAGGAGGCAGAGCACAG3’ | 5’GGTCGGCGTTTGGCTGAGAAGG3’ |
| ***BGLAP*** | **NM_199173** | 5’CCTCACACTCCTCGCCCTATTGG3’ | 5’TCGCTGCCCTCCTGCTTGG3’ |
| ***RUNX2*** | **NM_001024630** | 5’ACTGTCATGGCGGGTAACGATG3’ | 5’GTGAAGACGGTTATGGTCAAGGTG3’ |
| ***PPARG*** | **NM_015869** | 5’GGTTGACACAGAGATGCCATTC3’ | 5’TGGAGTAGAAATGCTGGAGAAGTC3’ |
| ***LPL*** | **NM_000237** | 5’CCGTGTGGCTCCAGAGTC3’ | 5’GAATGAGGTGGCAAGTGTCC3’ |
| ***FABP4*** | **NM_001442** | 5’AGAAGTAGGAGTGGGCTTTGC3’ | 5’ATCTAAGGTTATGGTGCTCTTGAC3’ |
| ***ACTB*** | **NM_001101** | 5’CTCTTCCAGCCTTCCTTCCT3’ | 5’AGCACTGTGTTGGCGTACAG3’ |
